# Perceptions of the double value coupon program in southern Illinois

**DOI:** 10.3389/fpubh.2023.1125069

**Published:** 2023-07-07

**Authors:** Dominique M. Rose, Saran Donahoo, Justin T. McDaniel, Dawn Null, Michelle McLernon, Aaron J. Kruse-Diehr

**Affiliations:** ^1^Nationwide Children’s Hospital, Center for Injury, Research and Policy, Abigail Wexner Research Institute, Columbus, OH, United States; ^2^School of Education, Southern Illinois University, Carbondale, IL, United States; ^3^School of Human Sciences, Southern Illinois University, Carbondale, IL, United States; ^4^Center for Rural Health-SMC, Southern Illinois University, Carbondale, IL, United States; ^5^Department of Family and Community Medicine, Univeristy of Kentucky College of Medicine, Lexington, KY, United States

**Keywords:** nutrition disparities, food environment, food access, facilitators and barriers, farmers market, stakeholder engagement

## Abstract

**Introduction:**

Purchasing produce at farmers markets represents one method by which individuals can purchase and have access to healthful and seasonal fruits. Despite the extension of nutrition assistance programs to local farmers markets, fruit and vegetables consumption has remained below the recommended guidelines, specifically in rural geographical locations.

**Statement of purpose:**

The purpose of the study was to explore the aspects of the Link Up Illinois Double Value SNAP Nutrition Incentives Program (DVCP) and its effects on food selection at rural farmers markets for individuals enrolled in nutrition assistance programs.

**Methods/approach:**

The current study uses a qualitative methodology in order to uncover barriers local health departments and farmers markets face to implementing the DVCP in their communities and to discover the perspectives of low-income individuals who utilize the DVCP. This paper explores the organizational and community member perceptions of the DVCP and its administration. Semi-structured interviews and one focus group were conducted with health educators from county health departments, DVCP stakeholders, farmers market managers, local farmers, and residents who used the DVCP. A purposeful sampling method was used, intentionally selecting individuals with lived experiences of the research objective. Data were analyzed using a three-cycle coding process, then categorized into overarching themes until thematic saturation was reached.

**Results:**

There were a total of 19 individuals who participated in the study. Five themes and four subthemes emerged from data analysis, including organizational capacity, exposure to the DVCP, purchasing power, DVCP advancements, and values.

**Conclusion/implications:**

These findings contextualize the facilitators and barriers of multiple stakeholders when implementing nutrition assistance programs at farmers markets. Other similar “double value” programs can utilize these lessons when seeking to increase participation of underrepresented populations at local farmers markets.

## 1. Introduction

Positive health outcomes, such as the improvement of body composition and reduced cardiovascular risk’ is associated with the consumption of fruits and vegetables. The 2020–2025 Dietary Guidelines for Americans recommends individuals to eat healthfully throughout the lifespan to reduce the risk of chronic disease. Yet, national surveillance data and other research (1–4) unfailingly indicate that low-income and rural populations are less likely than high income populations to reach the recommended guidelines for fruit and vegetable consumption (5). Federal, state, and local governments have implemented several programs for low income populations to address the challenges of eating healthfully, including the Special Supplemental Nutrition Program for Women, Infants, and Children (WIC), the Supplemental Nutritional Assistance Program (SNAP), and the Senior Farmers Market Nutrition Program (SFMNP), all of which are operated by local and state health departments.

Disparities in food access are highest in rural communities partly due to a sparse food retail environment, transportation issues (i.e., frequent lengthy travel distances), and household economic concerns (6). As such, smaller rural communities often lose access to local grocery stores, resulting in an unequal distribution of food sources (6, 7). There are many reasons why grocery stores leave rural communities, including the declining population and the inability to compete with larger grocery chains (6, 7). To promote more equitable access to healthful foods, nutrition assistance programs extend their benefits to include farmers’ markets purchases for fruits and vegetables by using electronic benefits transfer or (EBT). EBT is an electronic system that allows recipients of government assistance to pay for food using SNAP benefits (8). These programs provide “double value” farmers market coupons (9), which are incentives where SNAP recipients can receive double their assistance value for purchasing fresh fruits and vegetables. The additional assistance is intended to directly address barriers associated with cost and availability of fresh fruits and vegetables. These benefits provide individuals with the opportunity to establish connections with those who grow the produce (10), interactions that might positively impact food purchase behaviors, increase motivation to try new produce, and ultimately improve overall health (10, 11). Nevertheless, despite multiple psychological and physical benefits, research suggests WIC and SNAP recipients continue to underuse both farmers markets and double value coupon programs (11, 12). Studies on the use of farmers markets (13–15) among SNAP and WIC recipients (11–13, 16–19) have suggested multiple approaches to combat barriers to farmers market use, including identifying how SNAP and WIC recipients use their benefits, delineating facilitators and barriers associated with double value coupon programs and nutrition outreach, and developing direct interventions to address these concerns. To increase farmers market use among low-income populations, a greater understanding of individuals’ attitudes and perceived barriers to using these benefits is both necessary and warranted (20).

In Illinois, the Link Up Illinois Double Value SNAP Nutrition Incentives Program (colloquially, the “double value coupon program,” or DVCP) allows recipients of all three food assistance programs—SFMNP, SNAP, and WIC—to receive double the value of federal nutrition benefits spent at participating farmers markets throughout Illinois (21, 22). Although there are data regarding how much money recipients spend using the DVCP per month, it is still unclear where and how individuals spend their benefits, what local agencies see as impediments to implementing the program, and what barriers might keep recipients of nutrition assistance from using the DVCP in southern Illinois. Accordingly, the present study attempted to uncover barriers to implementing and using the DVCP in the predominantly rural communities of southern Illinois by (1) assessing the administrative scope of the DVCP from the perspective of organizational leaders such as community stakeholders, farmers, farmers market managers, and local health department administrators; and (2) exploring the self-reported usage patterns of low-income individuals who utilize the DVCP as well as their perceptions of barriers and benefits of program utilization.

## 2. Methods

### 2.1. Study design

In this qualitative study, data were collected using semi-structured interviews with various stakeholders who contributed to the operation of regional farmers markets across southern Illinois. Additionally, a focus group was conducted with individuals in Jackson County who have access to, and who use, the DVCP at the largest regional farmers market. This congressional district was selected purposively for its primarily rural location [i.e., median Rural–Urban Continuum Code (RUCC) of 4, indicating non-metro] and higher than average percentage of persons in poverty [average of 14.75% (range: 4.1–24.2%) across counties, compared to 12.1% in Illinois as a whole (21, 22)].

### 2.2. Data collection

All activities were approved by the human subjects committee at the institution where the research was conducted prior to engaging in study activities. Health educators from eight regional health departments with oversight of distributing WIC, SNAP, and SFMNP benefits to local residents were selected purposively (23, 24) from within the congressional district to participate in semi-structured interviews. To promote data triangulation, researchers also interviewed DVCP stakeholders (including the farmers market manager), a local farmer from the largest regional farmers market, and an administrator at the local cooperative grocer that accepts DVCP coupons. In addition, local residents who used the DVCP participated in a focus group to discuss their lived experiences with using the DVCP, to better contextualize emerging themes.

The Health Belief Model (25) (a value expectancy theory) was used as a guiding framework for the development of the interview guides and protocol. The lead author developed the interview guide, and the co-authors provided feedback until agreement was made and the interview guide was finalized. Data were collected using four versions of an investigator-developed open-ended qualitative interview protocol and demographic form: one for health department educators, one for local stakeholders, one for the grocery administrator, and another for focus group participants. Stakeholder interview questions measured participants perceived benefits and barriers of implementing the DVCP in their respective jurisdictions. Focus group questions probed participants’ readiness to purchase fresh and healthy food and their perceived benefits of using the DVCP. Individuals were contacted via telephone to schedule a time for face-to-face semi structured interviews. Written consent was established, interviews were conducted in the participants’ natural settings (e.g., office, farm, farmers market, co-op grocer). Eligibility criteria for the in-person interviews included (1) working at a health department in the 12th congressional district, and (2) held an administrative role in the WIC or nutrition division of the health department, (3) held an administrative role with the DVCP or farmers market. The focus group was conducted in a private space at the local cooperative grocer. Eligibility criteria for focus group participants included (1) Currently a SNAP recipient; (2) 18 years or older; (3) Able to read, write, and speak English; and (4) Have experience with the DVCP. Refreshments were provided, and participants were given a $10 incentive upon completing the focus group. One researcher (lead author) completed both the focus group and the in-person interviews. This researcher, at the time this research was conducted, has previous experience in conducting research examining farmers market use in urban communities and has been a part of community coalitions to assist in resource development for low-income communities. Individual interviews and the focus group were both, on average, 50 min long.

### 2.3. Data analysis

De-identified qualitative data were transcribed verbatim into a Microsoft Word document and were organized, managed, and coded using ATLAS.ti 8. Demographic data were analyzed using Statistical Package for the Social Sciences (SPSS) version 24. Two researchers participated in the coding of the qualitative data, the lead author and a paid external qualitative researcher. A three-coding cycle process was used as described by Saldana (26), using a combination of descriptive and *in vivo* coding. For the first cycle, data were entered into ATLAS.ti 8 using elemental coding ( 26). The first-to-second cycle consisted of eclectic, or open, coding to refine codes from the first cycle and break down data for interpretation (27, 28). Pattern coding, consisting of explanatory or inferential codes to help identify emerging themes or explanations (28), was used for the second cycle to further refine codes. Finally, data were analyzed and sorted, and codes were placed into categories to represent consistent and overarching themes. To promote interrater reliability and provide additional insight into theme development (27), an independent coder validated and independently coded the data during each phase of the coding cycle, and member checking was employed to ensure transcription fidelity and accuracy of interpretation. Like all qualitative research, interviewing is iterative in nature, for this study, data collection and analysis occurred simultaneously to explore if any new topics were introduced by participants (29). The authors agreed that saturation was reached when the analysis revealed no new categories or themes.

## 3. Results

### 3.1. Sample characteristics

A total of 19 individuals participated in the study. The average participant age was 42 years old (±17, range: 18–74). The majority were female (89.5%), white (68.4%), and college educated (63.2%), with 56.2% reporting full-time employment status. Of these participants, 11 were individuals with an administrative role in nutrition at a health department or DVCP, and farmers market, with an average age of 48 years old (±11, range: 33–69), most of whom were full-time employees (81.8%) with graduate professional degrees (54.5%). Focus group participants (*n* = 8) were, on average, 35 years old (±22, range: 18–74), female (87.5%), white (50%), employed part time (50%), and SNAP recipients (87.5%). Most had completed some college or technical school (62.5%). The remaining participants held positions at the farmers market (i.e., one farmer and one farmers market manager) or an administrative role with DVCP (co-op grocer). Table 1 displays these demographic data.

**Table 1:** Participant demographics.

Variable	*X*	%
Gender		
Female	17	89.5
Male	2	10.5
Race/Ethnicity		
White	13	68.4
Black or African American	4	21.1
American Indian or Alaska Native	1	5.3
Hawaiian Native or Pacific Islander	1	5.3
Employment status		
Full time	10	52.6
Part time	6	31.6
Unable to work	2	10.5
Not employed	1	5.3
Level of education		
College undergraduate degree	6	31.6
Graduate or professional degree	6	31.6
Some college or technical school	5	26.3
Some high school and GED certificate	1	5.3
6th Grade	1	5.3
Government benefits		
None	10	52.6
SNAP benefits	7	36.9
WIC benefits	2	10.5
Administrative Role		
Health department administrator	8	72.7
Farmers market, farmer and manager	2	18.1
DVCP administrator, local Co-op grocer	1	9.0

### 3.2. Organizational capacity

Data analysis of the focus group and one-on-one interviews revealed a total of five prominent themes, the first of which was organizational capacity, defined as factors local health department and farmers market administrators face when determining whether or not to implement the DVCP. Overall, participants identified numerous benefits of the DVCP. One local farmers market administrator suggested “continuing with the farmers market [despite approaching the end of grant funding for the DVCP]” (Health Department Administrator, PT06). Likewise, a health department administrator suggested “permanent funding” (Health Department Administrator, PT04) for the DVCP, and another mentioned “expanding the program to all counties or statewide,” (Health Department Administrator, PT05) indicating the need both to sustain and grow the DVCP. Nevertheless, numerous barriers to implementing the DVCP were cited, the most frequent being “governmental partnerships” at the regional, statewide, and federal levels, as well as those related to governmental programs such as SNAP and WIC. One participant identified “a need to build relationships and partnerships with the SNAP program and doctors’ offices [because] surprisingly, a lot of the doctors do not know what WIC is” (Health Department Administrator, PT01). Participants mentioned other needed partnerships, including “the development of a food council, [partnerships with] faith-based organizations, collaboration between doctor offices [and] grocery stores, and partnerships with the public housing authority” (DVCP Administrator, PT08). Participants strongly believed that building relationships and improving communication among multiple organizations would minimize nutritional barriers experienced by low income individuals (Table 2).

**Table 2:** Summary of interview questions for health department administrators, stakeholders, and DVCP recipients.

Interview questions – Health Department Administrators and Stakeholders
What do you think might be some ways to both *improve* and to *sustain* the food environment in your jurisdiction?
What community or state partnerships do you feel are necessary to improve the community nutrition environment for disparate communities or populations? Are any of these partnerships currently established? (If “yes”) Which ones? (If “no”) Why do you think they have not yet been established?
What do you know about the Double Value Coupon Program? What are your thoughts about it? Has your organization implemented a program similar to the DVCP? Have community members suggested implementation of the DVCP or any similar program?
Speaking now on overall community health, what specific ways does your organization improve the nutrition of the communities you serve? Do you feel that the DVCP would “fill a gap” to improve the community nutrition environment? (If “yes”) Could you give me some specific examples of ways you foresee that it might help? (If “no” or “it does not”) Why do you feel it would not improve the community nutrition environment? Specifically, what areas do you feel would be ineffective? What do you think are some of the benefits of *farmers markets* implementing the DVCP? For *shoppers* who use the DVCP, what do you think are some of the benefits for them?
What do you think are some barriers to exposing and/or expanding the reach of the DVCP to your community? What do you think would be the best way(s) to reduce those barriers? What are the demographics of individuals who seek out information about the DVCP? (Probing: Do they come from certain areas of town/the county? Do they share any particular demographic characteristics?) Are there any key demographic segments of the population that you think might not be adequately seeking out and/or receiving the benefits of the DVCP? (If “yes”) Why do you think that *difference* might exist? Are there individuals you think are receiving DVCP benefits but not *redeeming* them adequately? (If “yes”) What issues do you think might make it difficult for individuals to redeem their coupons? What do you think are some of the barriers to exposure and/or expanding the reach of the DVCP throughout Southern Illinois? What do you think would be the best way(s) to reduce those barriers and expand/better market the DVCP?
Interview Questions – DVCP Recipients
How do you think you personally benefit from using the DVCP? Tell me about any experiences you have had with the DVCP. What would you consider some of your *best* experiences? Could you describe them?
Tell me about any experiences you have had with the DVCP. How about disappointments using the DCVP? Could you describe those as well? What about the DVCP do you think needs improvement to make it better, more effective, or more useful to you and others who might benefit from it? Suppose you were in charge of making *just one* change to the DVCP that would make it better, and let us also assume that “money is no object.” What change would you make and why?
What are some factors that influence your decision to purchase fresh produce? How easy or difficult is it for you to get to places where you can shop for fresh produce? I’m referring specifically to transportation options. How does quality play a role in your decisions to shop for fresh produce? How does price influence your decisions to shop for fresh produce? What are your thoughts on the selection of fresh produce in the local community when shopping? Is access a significant factor when shopping for fresh produce? [If “yes”] Could you give me some examples of what you mean by *access*? About how often do you shop at the Carbondale Farmer’s Market or Carbondale Community Farmers Market at the Carbondale High School? What would you consider your greatest influences to shop at the farmers market? By *influences*, I mean people, things, or even emotions you might have.
Discuss your level of comfortability when using the DVCP at the market. Is it easy or hard to use? Could you give me some examples?

A subtheme of organizational capacity was (lack of) support. Farmers markets administrators experienced a limiting scope, one commenting, “We have frequent staffing issues that prohibit implementation, [such as] the uncertainty of funding [state and grant], and the lack of staff to effectively collaborate” (Health Department Administrator, PT06). Another administrator similarly noted, “I would love for my organization to offer a program such as the DVCP; however, it is hard fighting for programs with leadership inconsistencies and frequent staff changes” (Health Department Administrator, PT05). Community support was also a consistent attribute, with participants who held administrative roles describing the need for more individuals to take advantage of the DVCP. Finally, participants discussed the need for financial support, noting the instability of funding for the DVCP and the need to sustain the program with local funding through county partnerships and collaborations.

In some areas where health departments lacked capacity to implement the DVCP, they adopted similar approaches to reduce the nutritional gap in their communities. For example, in one county, a health department administrator collaborated with a local business owner to provide “fruit and vegetable dollars” to local community members and also partnered with two farmers to implement “Farmers Market Dollar Days” in the parking lot of her organization This individual also provided program education (about eligible food, in-season produce, and information about the farmers) in addition to providing recipes along with the premade produce bags for participants. These two elements reminded community members to engage in healthy eating activities, while providing awareness of the program and produce options at the market. Thus, although some participants may not have had the capacity or support to assist with the implementation of the DVCP, they nonetheless made efforts to improve community nutrition using grassroots methods.

### 3.3. Exposure to the DVCP

The second theme, exposure to the DVCP, was exemplified by participants’ experiences with the DVCP, whether they had participated in the program or simply had heard of it. It also encompassed their general experiences with fresh fruits and vegetables and familiarity with farmers markets. For example, one participant commented, “This program (WIC) has allowed participants to explore new fresh fruits and vegetables, learn about the farmers market, and discover the DVCP (Health Department Administrator, PT01).” One focus group participant, however, noted that some local residents may not have equal opportunity to experience this type of program:

Single mothers or working parents…don’t all have the luxury of waking up as early as 8:00 am to get the kids ready and go to the outdoors market. They unfortunately have to go to work or take their kids to a doctor’s appointment due to their schedules throughout the week (DVCP Recipient, PT13).

Similarly, advertisement of the DVCP was a sizable barrier. Participants with knowledge of the DVCP claimed that many individuals eligible to receive the program’s benefits remain unaware it even exists. For instance, two participants mentioned that area college students and individuals with lower incomes, two groups that could benefit from utilizing the DVCP, do not visit the farmers market as much as they might otherwise had they known of its existence.

Likewise, when discussing necessary improvements to the DVCP, participants mentioned the need for an increased social media presence to promote program expansion. One participant suggested, “They could make a Facebook page for it and promote it. The farmers market is on Facebook but does not post things like the [DVCP] program. They should work together and post together” (DVCP Recipient, PT14). Another participant agreed, stating, “I love Instagram, and they can promote it like a business or tweet! Twitter is fun and you can capture every second” (DVCP Recipient, PT17). In these instances, participants implied both the farmers market and DVCP were not collaborating enough to attract individuals to the market and were not using social media appropriately to communicate incentives and programming.

Participants also commented that the DVCP would “fill a gap” to improve community nutrition by increasing local access to fresh produce. They suggested that implementation of the DVCP program would likely result in a significant increase in fruit and vegetable consumption and, to overcome barriers, partnerships with local faith-based institutions and the formation of a food policy coalition—which would specifically “assess and address food access and issues”—could be established. Stakeholders suggested that community support would be critical to promote exposure for the DVCP. One participant argued that “strengthening community support for the program, whether it’s selling a bumper sticker or a reusable shopping bag or something like that, [would result in] proceeds [going to the DVCP]” (Farmers Market Manager, PT09). This specific participant urged the community to “step up and step in” to assist other community members by getting the word out so individuals could take advantage of the DVCP.

### 3.4. Purchasing power

Purchasing power was the third major theme identified and was reflective of DVCP recipients’ personal experiences when using the DVCP. One participant spoke about the many benefits of the program mentioning that “it helps put food in [her] fridge” (DVCP Recipient, PT12). Participants described their ability to purchase affordable, healthy food, because of the DVCP. Another DVCP recipient shared, “When I cook, I use that program; you know, it’s a real help… [with] a lot of things we cannot afford…It really helps” (DVCP Recipient, PT16). Three subthemes emerged from this theme: affordability, attractiveness, and variation. The subtheme of affordability related to DVCP recipients’ decisions regarding whether it made sense to purchase fresh produce. Overwhelmingly, participants listed price as the number one determining factor when making the decision to shop for healthy food. One explained, “If the price [were] really high, then I would not buy it. I would buy something less healthy” (DVCP Recipient, PT19). Another DVCP recipient spoke to affordability, mentioning:

(Price) is super-important, because if I don’t have enough money from my job or from my SNAP benefits… [and] I can’t afford the fresh fruits and vegetables, then I’m probably just going to be eating ramen for that time period (DVCP Recipient, PT14).

Some participants indicated they conflated attractiveness with quality and that they were less likely to purchase produce if they found it to be visually unappealing. One participant explained, “[Quality] is definitely important for me, especially for my fruits. I do not like bruises on any of them. I probably will not buy it if there are any bruises or if they look spoiled” (DVCP Recipient, PT14). Another participant spoke to the attractiveness or quality of the fresh fruits and vegetables, stating:

I think quality is really important because if a fruit or vegetable [is] gross, then I won't touch it or get it. I would have to go to another store just to find fruits and vegetables. Sometimes I don’t have the extra money to spend on gas to travel to another store. If I don’t have the extra money, then I won’t buy fruits or even vegetables that paycheck (DVCP Recipient, PT12).

The final subtheme is related to the assortment or variety of fresh produce in the local community. Focus group participants were asked, “What are your thoughts on the selection of fresh produce in the local community when shopping?” Participants were not impressed and debated whether their community might have an unfavorable selection and lack of variety. One participant stated, “I wish there [were] more access to different kinds [of fruit]. Like more or what’s wild, you know, [like] dragon fruit? Because sometimes strawberries may be a little boring, so you kind of want something different” (DVCP Recipient, PT17). Another noted, “…sometimes they do not have what I want, but I understand that it might not be in season, but it’s still always the same stuff” (DVCP Recipient, PT18).

### 3.5. DVCP advancements

Individuals spoke about program advancements related to incentive distribution. One participant suggested, “I kind of wish they would put it on the [LINK] card…They give us paper coupons, when they could just upload it onto the card” (DVCP Recipient, PT12). Another participant added, “We all use our phones… I know that she mentioned social media because we always get notifications from every app that we have, [but] why [does not] the program make an app instead of coupons” (DVCP Recipient, PT17)? Similarly, another participant agreed, “They should make an app for the market to track the program and the number of users; if people get a notification that the market is open and to visit certain stands that accept the market, they would be in big business” (DVCP Recipient, PT16)!

Further suggestions included expansion of the program to include additional benefits and added educational opportunities about the fresh produce. One participant mentioned, “I would give more—triple it instead of doubling the incentive. Maybe more people will use it” (DVCP Recipient, PT15). With regard to education, another participated stated, “I wish they would tell me how to make the vegetables that I’ve never cooked. Sometimes I want to buy the foods but do not want them to go to waste” (DVCP Recipient, PT19). For example, one innovative technique suggested was the use of meal kits. One participant claimed, “I would have them develop a cheaper meal-prep kit; it should at least be an option… I know there are ones at [local chain grocer], and they are almost $20, so if it [were] even half that price, that would be helpful” (DVCP Recipient, PT14). Another participant commented on how meal kits would promote ease of use, stating, “I do not have time to sit there and try to come up with a recipe or try to find a recipe, so if they had those meal kits, it would be a lot easier” (DVCP Recipient, PT17). The recommendations of education and recipe availability suggest that individuals may be more susceptible to cooking fresh produce if they receive more information at the time of purchase.

### 3.6. Values

The final theme, values, described participants’ normative beliefs regarding fresh produce, including preparation, cooking and shopping for fresh produce. One participant expressed a desire to bring back family traditions that had disappeared throughout the years:

My grandmother used to have this large garden in her backyard, and on Sunday mornings, we used to go in the back and pick fresh greens and vegetables for dinner. We don’t do that anymore, and I would like to start again. My first step in doing so would be purchasing fresh fruits and vegetables at the market. I want my family to get familiar with fresh vegetables early and one day be able to start our own garden in the back yard (DVCP Recipient, PT12).

Another participant mentioned how her sibling influenced her own shopping habits at the farmers market:

My greatest influence is my sister; she goes every year, more than twice a month. She cooks for her kids and husband with most of what she purchases at the farmers market. I think that seeing her be able to use the program and her telling me how much money she has saved influenced me to just visit (DVCP Recipient, PT19).

Finally, friends’ shopping behaviors were also highly influential in participants values related to the farmers market. One individual stated:

If it wasn’t for my friends dragging me out on a Saturday morning, I wouldn’t have learned about the farmers market, let alone the [DVCP] program. At first, I thought it would be a waste of time going there, but I’m glad I did. Saved money, they’re a good influence. We cook our healthy meals together too, and we go every weekend it’s a great experience (DVCP Recipient, PT17).

Collectively, these data suggest participants normative beliefs had a strong effect on behavior change, as such this direct influence from peers is needed to gauge individuals’ interest in both fresh and healthy food and the DVCP.

## 4. Discussion

This study explored barriers local health departments and farmers markets face when implementing or deciding whether to implement the DVCP in their communities, as well as the perspectives of DVCP recipients and how they presently use the program. Of the individuals interviewed, most had a minimum understanding of DVCP goals. Although administrators were well-informed of their community’s nutritional needs, they faced many organizational barriers, such as the lack of consistent leadership and employees to sustain the DVCP. In one instance, a participant collaborated with community members (i.e., farmers, local businesses, and her organization) to develop a program (similar to the DVCP) that offered discounted fresh fruits and vegetables, education, and “farm fresh” meal kits. Nonetheless, the organizational capacity of participants’ organizations varied considerably, thus ultimately determining what the participants were able to do regarding developing partnerships outside of their respective organizations.

Participants suggested that the development of partnerships in the respective counties were critical to improving the nutritional environment and increasing access to fresh produce, specifically grocery store collaboration and community support. One recent study revealed that a cooperative agreement between the consumer food environment (i.e., grocery store) and community members was a powerful component in improving the community food environment in rural or small counties (30). Ultimately, the development of programs within a community should begin with the community members themselves. In order for programs to be effective and reach their target populations, community members should inform every step of the development and implementation process to ensure programs are tailored specifically to community needs. Gupta (31) argues that grassroots programming must meet the needs of individuals who are typically economically disadvantaged and who would likely thus use their own knowledge to solve their own localized problems. Therefore, before community members can support a program, individuals from the community (including local university researchers) should be the initial contact and collaborators within any particular project. Building this sort of coalition should first begin with the gathering of community members, then building support outward to include surrounding community organizations.

Although local health department administrators mentioned that the DVCP would fill a gap to improve the community nutrition environment, one participant thought it was insufficient due to underuse by community members. Two barriers potentially exist which contribute to the underuse of the DVCP and farmers market use—marketing/promotion and education. Most participants revealed that word of mouth has been the primary reason for visiting the farmers market and learning about the DVCP. Focusing efforts on analyzing the target audience or potential DVCP recipients and promoting the program directly to individuals who qualify for the program could significantly impact DVCP use. The second barrier, education, plays a momentous role in individuals’ knowledge of the production and consumption of fresh fruits and vegetables. For instance, participants noted that they were interested in consuming more exotic fruits (e.g., dragon fruit) in southern Illinois. This comment implies that individuals might lack a fundamental understanding of both what local farmers can grow and the main concept of a farmers market, i.e., that consumers can buy directly (and locally) from the farmer. On the other hand, some participants commented that they wished to receive additional recipes and meal kits for in-season produce. Ironically, one of the local health department administrators offers both an education course (prior to the local farmers market/stand) with educational lessons on meal kits to their local patrons. Similarly, another participant’s concerns about the attractiveness of produce suggests that DVCP recipients may not fully comprehend the limitations of small independent farmers (compared, for example, with large grocery chains that often dispose of bruised or otherwise “imperfect” produce). Perhaps if the local rural farmers market offered extended educational outreach, pre-made fresh produce bundles, and recipes for “meal-kits,” the program might be more attractive to DVCP recipients.

The findings from this study should be interpreted with some limitations in mind. First, this study is limited in scope and used qualitative methods with a sample comprised of diverse individuals involved in local farmers markets who were sampled via convenience. Additionally, this study focused solely on one Congressional district, comprised of eight local health departments and two stakeholders involved in the DVCP in southern Illinois. To assess the administrative scope of the DVCP, participants were recruited from nine locations. Another limitation was whether organizational employees had the knowledge and experience in the subject of this specific inquiry. Some participants did not understand some questions presented and could not provide a response upon receiving clarification of the question. Furthermore, for the focus group, participants self-selected to participate in the study, and the condition of the weather during farmers market hours could have had an influence on participants’ ability or desire to sign up for the focus group. Given time constraints, only one focus group was conducted. Ultimately, the findings from this qualitative research study must be interpreted with caution and cannot necessarily be generalized to other geographical settings.

### 4.1. Implications for research and practice

To promote health equity, nutrition assistance programs are implemented in low income and rural communities to increase access to fresh and healthy and foods. Before the implementation of such programs, it is important first to conduct community assessments. Community assessments are generally performed by community-based researchers and practitioners to provide a method for examining strengths and resources, as well as concerns of a particular population or community (32). Community assessments are used for a variety of purposes and have been increasingly employed to examine food-related concerns in communities (30–32). More specifically, conducting a community food assessment—the examination of the local food system along the continuum of production to consumption, which includes growing, processing, distribution, and eating (33, 34)—would be most beneficial. A community food assessment can provide answers to questions about the ability of existing community resources to provide adequate and nutritionally-sound amounts of acceptable foods to households in the community (34), and the findings would likely give researchers and practitioners the opportunity for information exchange to determine what communities have, what they lack, and what they need. Furthermore, the collaborative nature of this approach provides opportunity for grassroots development of programs that could include permanent and engaged members of the community. This type of grassroots programming builds community support for a healthy, sustainable food system and holds promise to greatly reduce access barriers to healthy, affordable, and nutritious foods in all neighborhoods and regions (35, 36).

Lastly, researchers should consider using a systems-thinking approach to improve the community nutrition environment. Systems thinking helps researchers find the most important places for an intervention that might change the long-term behavior of a system. Using systems-thinking tools might help inform researchers, practitioners, and policy makers to ask the right questions toward understanding the best places to “leverage change” in a system. Leverage points include “places within a complex system… such as a company, program, [or] economy” “…where one small shift can produce big changes in everything” (37) (p.5). Currently, the DVCP and similar programs offer changes solely in the form of physical events, leverage points that would be considered “shallow” places consisting of interventions that are comparatively easy to implement yet bring about little change to the overall functioning of a system (as a whole) (37). Arguably, policy interventions and dominant scientific research must reinforce each other (37). In other words, more shallow interventions are often chosen in both research and policy, perhaps because of their ease of implementation. Our recommendation is that further research should focus on deeper issues of structures, values, and goals that shape the overall food system, especially in rural and underserved areas.

## 5. Conclusion

This study revealed that the DVCP is a valuable program not only to its recipients but also to local health department administrators, the latter of whom would be willing to implement the program given enough support and organizational capacity. As such, this study is the first step in better understanding what partnerships are needed between local farmers, farmers markets, and/or farm stands and local organizations to implement the DVCP and make it appropriately marketable to its intended constituents. Building capacity has the potential to improve the nutrition environment for lower-resource individuals who may not be able to access the DVCP. The results of this study can ignite future research that might ultimately influence policy to change organizational and political perspectives regarding solution-orientated change.

## Data availability statement

The original contributions presented in the study are included in the article/supplementary material, further inquiries can be directed to the corresponding author.

## Ethics statement

The studies involving human participants were reviewed and approved by the Southern Illinois University Carbondale Institutional Review Board. The patients/participants provided their written informed consent to participate in this study.

## Author contributions

DR conceptualized the study, conducted interviews, and focus groups, and served as primary author. SD and JM assisted with methodology and data analysis. DN and MM advised on nutrition-related and health department-related concepts, respectively including recruitment. AK-D was primary advisor for the study and contributed to authorship and led revisions. All authors contributed to revisions and approve of the final article.

## Conflict of interest

The authors declare that the research was conducted in the absence of any commercial or financial relationships that could be construed as a potential conflict of interest.

## Publisher’s note

All claims expressed in this article are solely those of the authors and do not necessarily represent those of their affiliated organizations, or those of the publisher, the editors and the reviewers. Any product that may be evaluated in this article, or claim that may be made by its manufacturer, is not guaranteed or endorsed by the publisher.
